# Postoperative Rhabdomyolysis in the Bilateral Shoulder Areas After Cardiac Surgery

**DOI:** 10.7759/cureus.18522

**Published:** 2021-10-06

**Authors:** Brian T Bueno, Pasquale Gencarelli Jr., Matthew H Nasra, Patrick S Buckley, James Monica, Brian M Katt

**Affiliations:** 1 Department of Orthopaedic Surgery, Rutgers Robert Wood Johnson Medical School, New Brunswick, USA

**Keywords:** bilateral, shoulders, surgery, postoperative, rhabdomyolysis

## Abstract

Rhabdomyolysis (RML) is a disease that results from the death of muscle fibers and the release of intracellular contents into the bloodstream as a result of traumatic or non-traumatic muscle injury. Postoperative RML is a rare complication that may result from improper patient positioning, extended surgery time, or unique patient risk factors. We describe a case of a 43-year-old obese male who presented with postoperative bilateral shoulder RML after undergoing cardiothoracic surgery for aortic valve disease. To our knowledge, after a thorough review of the literature using PubMed, Medline, and Google Scholar, no previous studies have reported positioning injuries specific to obese cardiac surgical patients and their relation to RML.

## Introduction

Rhabdomyolysis (RML) is a condition in which damaged skeletal muscle breaks down rapidly. Injury to myocytes results in disruption of the cell membrane and leakage of the intracellular contents into the extracellular space [1,2]. RML can result from both traumatic and non-traumatic etiologies. When RML occurs in the upper extremities, it is often due to strenuous exercise [3,4]. Postoperative RML is an infrequent complication that may result from a combination of improper patient positioning, extended surgery time, and patient risk factors such as a medical history of hypertension and diabetes [5-7]. We report a rare case of a 43-year-old obese male who developed postoperative bilateral shoulder RML after undergoing cardiothoracic surgery. The patient returned to baseline function after fluid resuscitation and close observation.

## Case presentation

A 43-year-old male with a past medical history of heart murmur since the age of 11, obesity (BMI = 39.6 kg/m^2^), hypertension, and aortic valve disease was admitted for elective cardiothoracic surgery. The patient had previously been diagnosed with heart failure and severe aortic insufficiency via transesophageal echocardiography. The patient subsequently planned to undergo reconstruction of the ascending aorta and replacement of the aortic valve.

On the day of surgery, the patient was in his usual state of health with no signs of infection. The patient had no musculoskeletal symptoms and had never had shoulder complaints or undergone shoulder surgery. In the operating room, the patient was placed under general anesthesia using propofol as an induction agent and remained supine in a Skytron surgical table for the entirety of the surgery lasting three hours and 36 minutes. The surgery included reconstruction of the ascending aorta and the proximal arch with graft and prosthetic aortic valve replacement, followed by sternal closure with wires and a plate. There were no intraoperative complications and the patient’s mean arterial pressure was maintained at 70 mmHg throughout the surgery. The patient did not suffer any spikes in temperature or have any episodes of increased muscle rigidity.

Two days following surgery, the patient complained of bilateral shoulder pain and was unable to raise his arms above his head due to weakness and pain. He denied ever having experienced similar symptoms before. The patient had been restricted to bed rest and was continuously monitored via telemetry on the cardiac floor postoperatively. Upon orthopedic consultation, the patient was afebrile, normotensive, and in no acute distress. The patient’s chest, back, shoulders, and upper extremities displayed no bruising or break in the skin and were soft to palpation. The patient had a full passive range of motion (ROM) but displayed weakness abducting against resistance in the bilateral upper extremities. The patient denied focal tenderness to bilateral upper extremities and sensation was intact to light touch in the distribution of bilateral median, ulnar and radial nerves. The patient did not display tenderness upon palpation of the cervical spine and did not have radicular symptoms.

**Figure 1:**
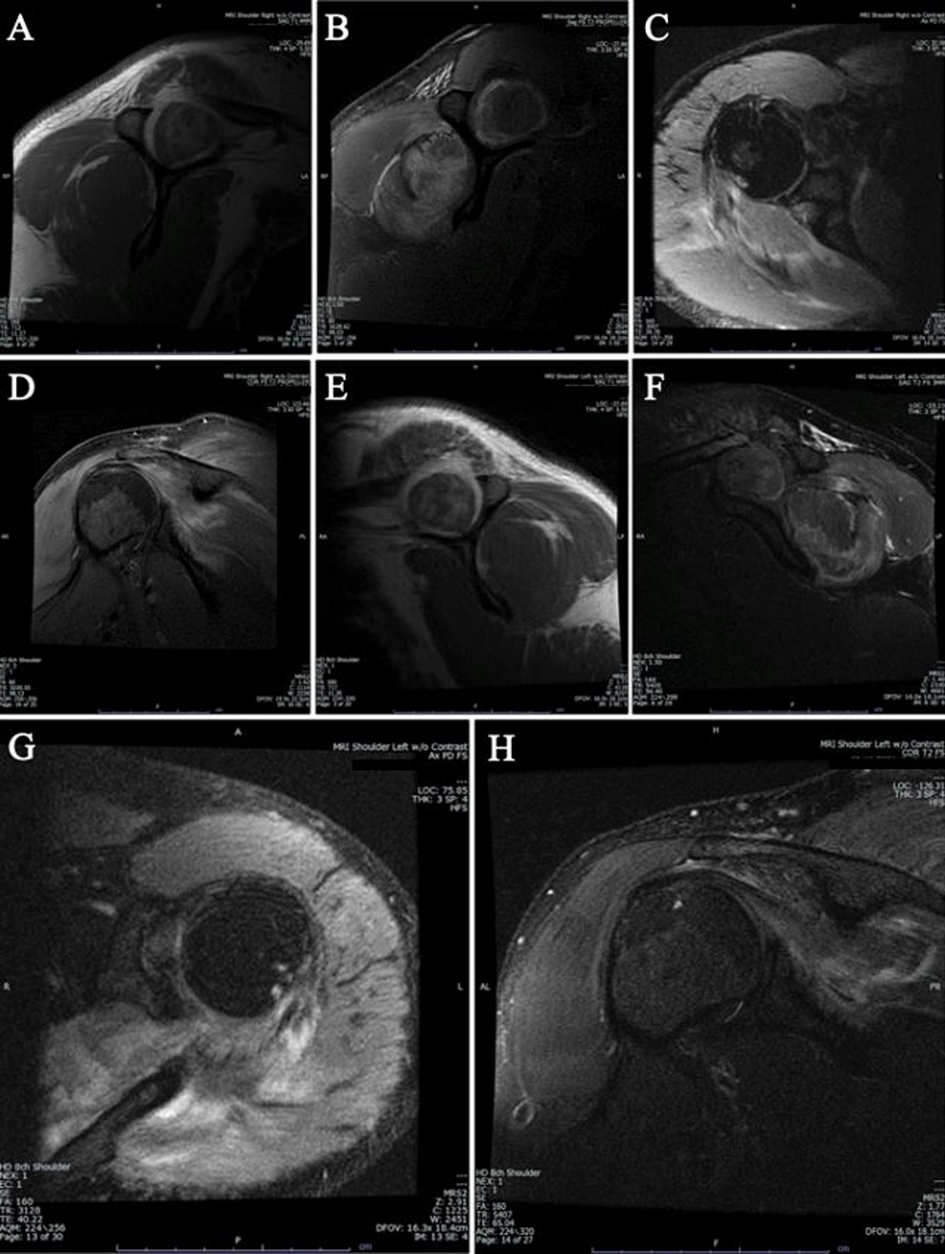
Postoperative bilateral shoulder rhabdomyolysis following cardiothoracic surgery confirmed with MRIs of bilateral shoulders without contrast. (A) Sagittal oblique T1-weighted MRI of the right shoulder. (B) Sagittal oblique T2-weighted MRI of the right shoulder. (C) Axial MRI of the right shoulder. (D) Coronal oblique T2-weighted MRI of the right shoulder. (E) Sagittal oblique T1-weighted MRI of the left shoulder. (F) Sagittal oblique T2-weighted MRI of the left shoulder. (G) Axial MRI of the left shoulder. (H) Coronal oblique T2-weighted MRI of the left shoulder. MRIs in multiple planes of bilateral shoulders demonstrate intramuscular hemorrhage involving the supraspinatus muscles bilaterally, prominent intramuscular edema of the infraspinatus, and teres minor muscles bilaterally, and mild edema in the posterior deltoid and trapezius muscles bilaterally.

The patient’s creatine phosphokinase (CPK) level was checked two days postoperatively and noted to be 14,990 U/L (normal range male: 60-400 U/L; normal range female: 40-150 U/L) with a blood urea nitrogen (BUN) of 31 mg/dL (normal range: 6-23 mg/dL), creatinine of 0.9 mg/dL (normal range: 0.5-1.2 mg/dL), and potassium of 4.7 (normal range: 3.5-5.0 mmol/L). The patient subsequently received additional intravenous (IV) hydration. Magnetic resonance imaging (MRI) of the bilateral shoulders showed intramuscular hemorrhage involving the bilateral supraspinatus muscles (Figures 1A-1H). There was also prominent intramuscular edema of the infraspinatus and teres minor muscles bilaterally, with mild intramuscular edema in the posterior deltoid and trapezius muscles bilaterally. These findings in addition to a markedly elevated CPK level were consistent with a diagnosis of RML. Of note, our patient was on Lovenox (Enoxaparin), 40 mg SQ daily which is standard of care for a patient after this type of cardiac surgery.

The patient was treated with a continuous IV infusion of normal saline for six days. His CPK levels consistently trended downward from 14,990 U/L on postoperative day 2 to 127 U/L by postoperative day 12; creatinine levels remained within normal limits and BUN levels were slightly elevated throughout the postoperative course (Table 1). Physical therapy was ordered and the patient performed ROM exercises which aided in alleviating his functional restriction. Additionally, analgesics were given for pain. By postoperative day 12, the patient’s pain and weakness had completely resolved and there was no clinical or laboratory evidence of persistent RML.

**Table 1:** Postoperative CPK, creatinine, and BUN trends Reference ranges: CPK male (60-400 U/L); creatinine (0.5-1.2 mg/dL); BUN (6-23 mg/dL). CPK levels returned to within normal limits at postoperative day 12. Creatinine levels remained within normal limits and BUN levels were slightly elevated throughout the postoperative course. CPK - creatine phosphokinase, BUN - blood urea nitrogen

	Post-Op Day 2	Post-Op Day 4	Post-Op Day 5	Post-Op Day 6	Post-Op Day 8	Post-Op Day 12
CPK (U/L)	14,990	5,451	3,531	1,315	398	127
Creatinine (mg/dL)	0.9	0.7	1.0	1.1	1.1	1.2
BUN (mg/dL)	31	26	24	23	26	24

## Discussion

RML is characterized by the breakdown of muscle tissue and release of muscular cell contents into the circulation, which may be caused by exertional injuries, crush injuries, ischemia, extremes in body temperature, drugs, and toxins, metabolic disorders, or infections [1,2]. A fivefold elevation of serum CPK levels is considered diagnostic for RML (males: CPK > 1,160 U/L; females: CPK > 1,075 U/L) and myoglobinuria on urinalysis (UA) is pathognomonic for the condition [2]; our patient did not have a UA performed. Other pathophysiologic sequelae of RML include acute tubular necrosis, hyperkalemia, hypocalcemia, hyperphosphatemia, hypovolemia, hypoalbuminemia, and possibly disseminated intravascular coagulation [2]. Compartment syndrome is a known complication that may arise from RML resulting in elevated intracompartmental pressures requiring emergent fasciotomies to prevent persistent neurological deficits, limb amputation, and even death [8].

Cases of exercise-induced RML of the upper extremities including the rotator cuff in well-conditioned athletes have been reported in the literature [3,4]. Causes of non-traumatic RML such as sedative medications or infection have also been reported [2,9]. Our patient demonstrated no clinical signs of infection. Because the patient received propofol for induction of anesthesia, he may have been at risk of propofol infusion syndrome (PRIS). Propofol inhibits and inactivates several enzymes in cell metabolism. Therefore, cells that undergo high rates of metabolism, such as skeletal muscle, are at risk of damage during propofol infusion. PRIS often presents with RML, hyperkaliemia, acute kidney injury, cardiac dysfunction, and high rates of mortality during or immediately following propofol infusion [9]. Our patient is unlikely to have developed PRIS considering his presentation two days after propofol infusion and lack of additional signs and symptoms associated with PRIS. Our patient’s isolated RML is more likely a result of patient positioning.

Moreover, patient positioning and extended surgical time are known risk factors for postoperative RML [7]. In the operating room, improper patient positioning and/or insufficient padding can result in both crush injury and hypoxia to skeletal muscle and subsequent RML. Obese patients are more prone to pressure-related injury when improperly positioned in surgery, particularly in the gluteal and shoulder regions [5]. Most cardiac procedures occur in the supine position, allowing for increased weight distribution, yet the gluteal and shoulder areas are still at risk in obese patient [5]. Padding of pressure points including the gluteal, shoulder, and lumbar regions that contact the operating table has been advocated to decrease the rate of RML, but this may be counterintuitive as padding increases the force per unit area and is reflected in the continuing high incidence of RML [6]. In this case, the development of postoperative RML may be attributed to a combination of supine positioning, extended duration of surgery, and obesity. 

Chakravartty et al. performed a systematic review investigating the occurrence of RML after bariatric surgery [6]. The authors compared 87 patients with RML to 325 patients without RML who underwent bariatric surgery and concluded that patients who are male, have a BMI > 50 kg/m2, and underwent prolonged surgery (225 vs 207 min, p < 0.01) are at greater risk of pressure injury and subsequent RML [6]. Mognol et al. performed a study to determine the incidence of RML following laparoscopic obesity surgery in 66 patients and identified massive obesity (BMI > 60 kg/m2) and long duration of the operation (390 vs 110 min) as risk factors for developing RML [10]. Our patient’s male gender, BMI (39.6 kg/m2 ), and prolonged operative time (3 hours and 36 minutes) encompass risk factors indicative of developing postoperative RML.

Furthermore, Segaran et al. described the occurrence of bilateral lower leg RML in a 46-year-old obese woman status post bilateral breast reconstruction following chemotherapy for breast cancer [11]. The surgery was performed in the supine position under prolonged operative time (14 hours). The patient's obesity and reduced tissue perfusion secondary to her low preoperative hemoglobin (9.8 g/dL) were also cited as risk factors for developing RML [11]. Our patient presented with severe RML (CPK > 10,000 U/L) [11] with a CPK of 14,990 U/L on postoperative day two likely caused by a combination of related myocardial injury from cardiac surgery, prolonged operative time, and muscle ischemia secondary to obesity and supine positioning. However, our patient’s preoperative hemoglobin was 15.4 g/dL, suggesting tissue ischemia was likely related to intraoperative pressure injury decreasing blood flow to the shoulders.

Cardiac and aortic surgery has been suggested to put patients at risk of developing RML. Omar et al. describe cases of RML in patients who underwent coronary artery bypass grafting, valvular surgery, and aortic dissection repair [12]. The authors attribute the causes of these cases to possible hypoperfusion, patient positioning, and increased operative time [4]. Anthony et al. reported the development of paraspinal muscle RML in a 68-year-old male after open aortic reconstruction for an aortic aneurysm [13]. The authors proposed hypoperfusion secondary to hypothermic circulatory arrest compromised the lumbar arteries which supply the paraspinal muscles leading to RML [13]. Our patient underwent 125 minutes of cardiopulmonary bypass with resultant mean arterial pressure of 70 mmHg and body temperature of 24 C (75.2 F) but developed bilateral shoulder musculature RML rather than paraspinal RML. 

Brunette et al reported a case of postoperative bilateral plexopathy following a 5.5-hour laparoscopic bariatric surgery in a 39-year-old morbidly obese male [14]. The patient developed symptoms representative of brachial plexopathy including both motor and sensory deficits, loss of reflexes, and pain in both arms posteriorly. The authors contributed the etiology of the brachial plexopathy to the 40º head-up position of the patient during surgery without specific support of the minimally abducted bilateral arms (< 60º) resulting in caudal stretch injury [14]. In contrast, our patient’s surgery was performed in the supine position with no inclination. Moreover, although our patient displayed the initial weakness of the bilateral shoulders against abduction two days following surgery, he retained sensation to light touch in the C5-T1 dermatomes and had intact reflexes of the bilateral upper extremities. Thus, the combination of our patient’s surgical positioning, isolated weakness of the bilateral shoulders with intact sensation and reflexes, elevated CPK levels, and MRI finding diagnostic of rotator cuff intramuscular hemorrhage favor the diagnosis of RML over brachial plexopathy.

The best methods for the prevention of RML are yet to be identified [6], but increased intraoperative fluids do not seem to prevent RML or progression to AKI [15]. Cornerstones of AKI prevention include early IV hydration, urine alkalinization, forced diuresis, and correction of metabolic derangements [13]. Our patient received early and aggressive IV hydration postoperatively which most likely prevented progression to AKI and resolved his symptoms. Our patient's renal function was not impaired as his creatinine remained within normal limits and his BUN was only slightly elevated throughout his postoperative course. Due to his recovery with IV fluid hydration and clinical improvement, no muscle biopsy was performed to evaluate for a possible underlying metabolic abnormality that may have predisposed him to RML development.

The unique case presented above displays the occurrence of postoperative RML of the bilateral shoulders in an obese male following cardiac surgery. Although RML has been documented as a postoperative complication associated with procedures in the obese population, it has never been described as affecting patients’ bilateral shoulder musculature related to surgical positioning in obese patients undergoing cardiac surgery [5,6,10,12,15,16].

## Conclusions

With the increasing rates of obesity and secondary comorbidities leading to the need for cardiac surgery, providers should be vigilant for the unusual diagnosis of RML secondary to supine patient positioning in obese individuals’ status post cardiac surgery. If consulted for post-operative shoulder pain in a patient with risk factors as outlined above, the orthopedic surgeon should be aware of this rare diagnosis and begin appropriate treatment with aggressive IV fluid hydration. Routine CPK measurements, as well as close monitoring of clinical symptoms and renal function, should be initiated early to avoid end-stage renal disease, limb amputation, or death caused by the sequelae of RML.
